# Nonhuman Primate Models of Zika Virus Infection and Disease during Pregnancy

**DOI:** 10.3390/v13102088

**Published:** 2021-10-16

**Authors:** Nicole N. Haese, Victoria H. J. Roberts, Athena Chen, Daniel N. Streblow, Terry K. Morgan, Alec J. Hirsch

**Affiliations:** 1 The Vaccine & Gene Institute, Oregon Health and Science University, 505 NW 185th Ave, Beaverton, OR 97006, USA; haese@ohsu.edu (N.N.H.); streblow@ohsu.edu (D.N.S.); 2 Division of Reproductive & Developmental Sciences, Oregon National Primate Research Center, 505 NW 185th Ave, Beaverton, OR 97006, USA; robertsv@ohsu.edu; 3 Department of Pathology, Oregon Health and Science University, 3181 SW Sam Jackson Park Rd, Portland, OR 97239, USA; chenat@ohsu.edu (A.C.); morgante@ohsu.edu (T.K.M.); 4 Division of Pathobiology & Immunology, Oregon National Primate Research Center, 505 NW 185th Ave, Beaverton, OR 97006, USA; 5 Department of Obstetrics and Gynecology, Oregon Health and Science University, 3181 SW Sam Jackson Park Rd, Portland, OR 97239, USA

**Keywords:** nonhuman primate, zika virus, pregnancy, placenta, immune response

## Abstract

Since the explosive outbreak of Zika virus in Brazil and South/Central America in 2015–2016, the frequency of infections has subsided, but Zika virus remains present in this region as well as other tropical and sub-tropical areas of the globe. The most alarming aspect of Zika virus infection is its association with severe birth defects when infection occurs in pregnant women. Understanding the mechanism of Zika virus pathogenesis, which comprises features unique to Zika virus as well as shared with other teratogenic pathogens, is key to future prophylactic or therapeutic interventions. Nonhuman primate-based research has played a significant role in advancing our knowledge of Zika virus pathogenesis, especially with regard to fetal infection. This review summarizes what we have learned from these models and potential future research directions.

## 1. Zika Virus Emergence

Zika virus (ZIKV) is a mosquito-borne flavivirus that emerged as a major public health threat in South and Central America in 2015–2016. ZIKV was initially isolated from a sentinel rhesus macaque in 1947 in the Zika Forest of Uganda [1]. Until 2007, ZIKV had circulated silently in many parts of Africa and Asia without causing severe disease or large outbreaks, with fewer than 20 documented human infections [2]. However, large outbreaks (in what were presumably immunologically naïve populations) were detected on the Micronesian island of Yap in 2007, and in French Polynesia in 2013. These outbreaks preceded the introduction of the virus into Latin America in 2014–2015. ZIKV is primarily transmitted by Aedes species mosquitoes [3]. In addition, ZIKV infections acquired via sexual transmission and blood transfusion have been described [4,5]. In healthy adults, infection is usually asymptomatic, but may result in a mild, self-limiting febrile illness, characterized by a rash, headache, myalgia, and conjunctivitis (reviewed in [6]). However, during the recent epidemics, ZIKV infection was associated with more severe disease, including Guillain-Barré syndrome (GBS) in adults and congenital malformations in the fetus of infected pregnant women (e.g., microcephaly and fetal demise) [7,8,9]. Although the number of suspected/confirmed ZIKV cases in the western hemisphere has dramatically declined since its height of >650,000 in 2016, the virus remains present, with approximately 20,000–35,000 cases/year from 2018 to 2020 [10]. Additionally, sporadic outbreaks of ZIKV infection continue to occur in Africa and Asia [11].

The link between ZIKV infection and neurological abnormalities, now recognized as congenital Zika syndrome (CZS), in neonates is well established [12]. CZS comprises a spectrum of phenotypes, ranging from congenital microcephaly to more subtle phenotypes that manifest later in life. These include ocular and auditory abnormalities, cognitive and motor deficits, and seizures. Cohort studies attempting to determine the risk of adverse outcomes following congenital infection have reported widely varying estimates. Data from Rio de Janeiro, Brazil, described adverse outcomes from ZIKV infection in 46% of pregnancies [7], while data collected from the US and US territories show an adverse outcome rate of 5–14% [13,14]. These discrepancies may be due in part to regional differences in ZIKV diagnosis or reporting. Nevertheless, it is possible that the full impact of the ZIKV outbreak will not be appreciated until those born to infected women approach adulthood. 

## 2. Nonhuman Primate Models of ZIKV Infection and Disease

Nonhuman primates have been, and continue to be, integral to understanding the spectrum of phenotypes associated with congenital ZIKV infection. ZIKV infection has been extensively modeled in several species of nonhuman primates (NHP). The pathology of ZIKV infection in the adult macaque is generally limited to a transient rash, conjunctivitis, and fever. Viremia (as assessed by viral RNA or infectious virus) is observed as early as one day post-infection (dpi), following sub-cutaneous infection, and is usually resolved by 10 dpi. During this time period, ZIKV RNA can also be detected in the urine, saliva, and lacrimal fluid, as well as cerebral spinal fluid, and semen or vaginal swabs, which indicates that the virus is capable of quickly developing a widespread infection [15,16,17,18]. This finding is corroborated by detection of the viral RNA in multiple tissue types, including lymphoid, digestive, cardiopulmonary, musculoskeletal, genitourinary tract, nervous, and endocrine [16,17,19]. The infectious virus can generally be isolated from tissues with high viral RNA levels during the viremic period. While viral loads are highest shortly following peak viremia, the virus can persist in certain tissues for months. Notably, viral RNA was detected in primary and secondary lymphoid tissue, and found to persist in the lymph nodes of some animals through 72 dpi. Other areas from which viral RNA was detected include the stomach and regions of the small and large intestines, muscle and joint tissue, and tissues of the urogenital tract in males and females. 

As with humans, infection of pregnant female macaques has resulted in a range of fetal outcomes. Fetal loss occurs at a four-fold higher rate in ZIKV-infected rhesus macaque dams when compared to ZIKV-unexposed animals housed at the same locations [20]. In surviving NHP fetuses (rhesus and pigtail macaques, and squirrel monkeys), delivered near or at term, neurological pathology similar to that found in human fetuses has been observed. Histopathologic findings in the brains of these animals include calcifications, loss of neuroprogenitor cells, gliosis, vasculitis, lissencephaly, loss of ventricular ependyma, and reduced brain size [21,22,23,24,25,26,27,28]. Frank microcephaly, defined as head circumference < 2 standard deviations below aged matched controls, is occasionally observed. Notably, microcephaly in humans is sometimes inapparent until several months after birth, indicating that neuronal damage may result in continuing developmental abnormalities and delays [13,29]. Studies of neonatal rhesus macaques born to ZIKV-exposed mothers described phenotypes ranging from clinically inapparent to severe, including seizures, tachypnea, reduced muscle tone, and cardiomegaly. Histopathological evaluation of neonatal brain and tissues collected 17 d post-delivery yielded findings similar to those found in fetuses examined at delivery, even in cases in which clinical abnormalities had not yet manifested.

## 3. ZIKV Infection of the Placenta

Although the mechanism of ZIKV-induced fetal injury remains unclear, a combination of factors is likely involved. Infection of fetal neuronal cells, especially neural progenitor cells, clearly drives many of the pathologies associated with CZS. Indeed, post-natal infection of infant rhesus macaques (~37 days old) resulted in neurotropism and central nervous system abnormalities similar to those associated with congenital infection [30]. 

Additionally, ZIKV-induced placental damage and inflammation are frequent findings post-ZIKV infection, suggesting that this may also contribute to adverse pregnancy outcomes. NHPs are a highly relevant and suitable animal model for translational studies of placental structure, function, and response to infection. The placenta of mammals is classified based on criteria of gross shape, histological structure, and composition of the maternal to fetal transfer barrier [31,32]. The human placenta is discoid in shape, and is deeply invasive in the maternal uterine wall, leading to a hemochorial classification, where maternal blood comes in direct contact with the fetal chorion. Placentation varies across different animal species with primates, rodents, and rabbits being most closely related to humans. Further categorization at the cellular level is based on the presence of one (hemomonochorial), two (hemodichorial), or three (hemotrichorial) trophoblast cell layers found in primates, rabbits, and small rodents (mice and rats), respectively. In addition to the similarities of placentation, the NHP has a long gestational period of 6 months, shares key aspects of hormonal regulation, and has a comparable fetal developmental ontogeny to humans [33]. 

Data generated from in vitro model systems in combination with animal studies have greatly advanced knowledge of the effects of ZIKV infection of the placenta in the past five years. In particular, much has been learned about the timing of infection and susceptibility of different cell types that allow maternal to fetal transmission across the placental barrier. The uteroplacental environment is immunologically privileged [34,35,36], and TORCHs(z) (Toxoplasmosis, Other, Rubella, Cytomegalovirus, Herpes, Syphilis, and now Zika virus) infections are common at this interface [27,37,38,39,40,41,42,43,44,45] (Table 1). Although it is overly simplistic to equate pregnancy with immune suppression, certain immune cell subsets, generally associated with an anti-inflammatory phenotype, are prevalent in circulation, and in the placental and uterine environment. In the human placenta, a large proportion of the decidua is comprised of leukocytes (~40%), with decidual natural killer cells (dNKs) representing a majority of the cells (~70%), followed by decidual macrophages (20–25%), and T cells (3–10%) [46]. During normal pregnancy, there is a general bias toward T-helper (Th)2 cells compared to Th1, as well as an increase in the population of regulatory T cells (Treg) [47]. Within the decidua, the CD4+ T cells Th2 phenotype is promoted by IL-13, Il-10, IL-4, and IL-6 produced by placental supporting cells [48]. Further, placental macrophages (both decidual macrophages and villous stromal macrophages) skew towards an M2 phenotype (CD163+, anti-inflammatory, and Th2-promoting) upon the completion of placental development [49,50]. This regulatory phenotype is believed to be influenced by M-CSF and IL-10 secreted by trophoblasts. 

ZIKV infects maternal decidual macrophages, mesenchymal cells, immature endothelial cells, placental trophoblasts, and placental villous stromal macrophages (Hofbauer cells) at the uteroplacental interface [55,63]. In the context of ZIKV infection, there is an increase in the number of pro-inflammatory M1 macrophages, as well as a significant increase in the number of activated (CD169+) monocytes. Additionally, proliferation of CD4+ T-cells is increased in ZIKV-infected placentas compared to uninfected controls [27]. Early pregnancy infections are more likely to affect the placenta due to the predominance of replicating cytotrophoblastic anchoring columns, which attach the placenta to the uterine decidua, and the pro-angiogenic environment in both the maternal uterine arteries and fetal placenta [7,64,65,66]. The syncytiotrophoblast monolayer is a formidable obstacle to vertical viral transmission [34,36]. Viral replication in the syncytiotrophoblast layer is inhibited, but viral transport through this layer to the underlying susceptible cytotrophoblasts does occur, although less efficiently later in gestation [7,63,65]. In contrast, replicating cytotrophoblasts in the anchoring columns and immature proliferating endothelial cells are more susceptible to viral infection [7,64,65,66]. Once past the contiguous syncytiotrophoblast barrier, the virus is free to replicate in stromal Hofbauer cells [43,45] and fetal endothelial cells in developing villi [67,68]. 

ZIKV is about twice as likely to infect the placenta early in pregnancy compared to later gestational ages [7,65]. There is a range of reported uteroplacental findings, including Hofbauer cell hyperplasia [27,43,44,45,55], chronic histiocytic intervillositis [42], placental infarctions, and increased numbers of villous stromal calcifications [27,45]. Variation in pathologic findings between studies could be related to the timing of infection, viral load, and viral strains. Vasculitis is common in systemic viral infections, and maternal decidual leukocytoclastic vasculitis associated with ZIKV has been reported [27,44]. The susceptibility of pro-angiogenic immature endothelial cells [67,69], proliferating as part of pregnancy-induced uteroplacental vascular remodeling, suggests decidual vasculitis may be more ubiquitous than currently appreciated. 

One of our research group’s focused objectives has been to use advanced noninvasive imaging modalities to understand ZIKV in utero. Specifically, we have employed ultrasound and magnetic resonance imaging (MRI) methodologies to make novel observations of how ZIKV alters placental structure and subsequently impacts function. Our data have demonstrated perturbed maternal perfusion of the placental intervillous space with increased vascular resistance, despite an overall decrease in blood flow. This finding is correlated to an increase in fibrin deposition and decreased lumen diameter in the spiral arteries. In addition, we demonstrated an increase in villous damage with histological evidence of infarctions and calcification, macrophage activation, and increased cytokine expression, all of which correspond to the reduced oxygen permeability demonstrated by our blood oxygenation-dependent MRI data [27]. 

Endothelial cell damage in maternal uterine arteries would impact uteroplacental blood flow [27]. Loss of placental villous endothelial cells would lead to stromal cell death necrosis, and mineralization (calcifications) [70]. Although the fetal blood vessels and stromal cells die, the surrounding cytotrophoblasts and syncytiotrophoblast layer remain intact due to maternal nutrient supply from the intervillous space and relatively increased resistance to oxidative stress. This is a normal process in placental membrane formation at the end of the first trimester [70], but it is pathologic in chorionic villi essential for maternofetal exchange. Villous calcification is not specific for ZIKV infection, because it also occurs as the placenta ages, and in placentas from obese mothers on high-fat diets [71]. 

**Figure 1:**
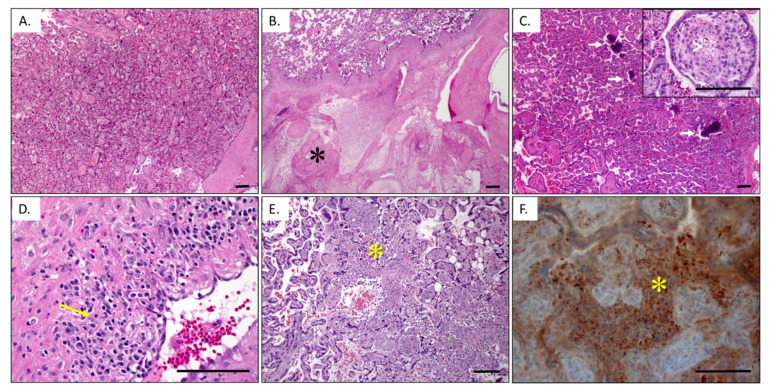
Zika related uteroplacental pathology. (**A**) Gestational age-matched negative control placental histology. In contrast, Zika infected placentas (**B**,**C**) are more likely to have lobular infarctions (*) and villous stromal microcalcifications (arrows). Early stages of villous stromal cell death are also seen (inset arrowhead). (**D**) Some cases have maternal decidual leukocytoclastic vasculitis composed of lymphocytes, plasma cells, and eosinophils (arrow). (**E**) Preliminary data in NHP models suggest a potential relationship with chronic histiocytic intervillositis (*), which is supported by CD68-positive macrophages (*) in the intervillous space around viable villi (**F**). Photomicrographs of hemotoxylin- and eosin-stained sections (**A**–**E**), as well as immunohistochemical stained section with hematoxylin counterstain. Bar is 100 µm.

In nonhuman primate models of ZIKV infection [24,27,44], maternal uterine vasculitis is a conspicuous feature along with placental infarctions and villous stromal calcifications (Figure 1). There is pronounced leukocytoclastic vasculitis in cases infected early in pregnancy (30–50 days of gestation, where term is ~168 days), with a mixture of lymphocytes, plasma cells, and a few eosinophils infiltrating the decidual spiral artery muscular wall. This type of vasculitis is usually related to hypersensitivity reactions, but viral infections are also a well-recognized cause of hemorrhagic leukocytoclastic vasculitis in the skin [68]. In our experiments, spiral artery vasculitis was absent in cases infected later in gestation (115 days of gestation), and these late exposures had fewer plasma cells in the decidua, fewer placental villous calcifications, and only microscopic placental infarctions [27]. We suspect the differences observed between early and late Zika infections may be related to differences in tissue susceptibility (less angiogenesis and no proliferating cytotrophoblast anchoring columns) [63,64,65], or the shorter timeframe between infection and tissue collection for analysis. 

An alternative working hypothesis may be related to fetoplacental metabolic demand versus maternal nutrient supply. An infected placenta would be expected to have increased metabolic demands that would be more likely to develop oxidative stress and cell death. The patchy, rather than diffuse, presentation of placental pathology argues against increased fetal demands. Moreover, we think the metabolic demand hypothesis is less likely because fetoplacental demands increase with gestation, which is why gross and histologic features of maternal vascular malperfusion are much more common in the third trimester compared with early pregnancy [72]. 

## 4. Maternal and Fetal Immune Response to ZIKV Infection 

To evaluate the maternal immune response post-ZIKV infection, researchers have primarily utilized blood samples and isolated PBMCs collected at various dpi. It is important to note that due to the limited amount of data on the maternal immune response post-ZIKV infection, the results described herein are a summation of multiple studies utilizing different NHP species, virus strains, and infection time points ranging from the first to the third trimester [21,22,24,27,73]. Any significant effect of these variables is emphasized. Using phenotypic analysis of PBMCs by flow cytometry with antibody panels designed to identify immune cell populations, CD16+ NK cells, monocytes, macrophages, and myeloid DC cell populations were shown to be activated (CD169+) by 5 dpi, with prolonged activation in some animals to 85 dpi [27,73]. CD8+ T cell proliferation, measured by Ki67 staining, is also increased after ZIKV infection, peaking from 5–14 dpi for effector memory (Tem) and central memory (Tcm) subsets with a second peak at 56 dpi for Tem and Tcm subsets [21,27,73]. Activated MR1 Tet− CD69+ CD8+ T cell numbers were also elevated between 2 and 7 dpi [21]. B cell proliferation was maximal between 10 and 14 dpi [27]. In a study by Nguyen et al., the number of circulating plasmablasts increased more slowly when animals were infected in the third trimester versus infection during the first trimester, but the overall endpoint response did not differ between first or third trimester infection [73].

The most studied aspect of the ZIKV specific maternal immune response is the development of virus neutralizing antibodies. Multiple studies have identified the presence of neutralizing antibodies in pregnant animals with initial detection ranging between 6 and 18 dpi and peaking at an average of 14–21 dpi [21,22,23,24,27,73]. Nguyen et al. observed at 28 dpi that pregnant animals developed neutralizing antibody profiles similar to ZIKV-infected nonpregnant animals, regardless of the trimester of infection. In contrast, rhesus macaques infected between 42 and 98 days of gestation (strain Paraiba 2015) showed substantially higher neutralizing antibodies eight weeks post-infection as compared to infection-matched nonpregnant females [24]. Neutralizing antibodies have been described as an important immune correlate of protection against ZIKV infection. In nonpregnant NHPs, rechallenge and vaccine experiments have demonstrated the protective capacity of neutralizing antibodies; antibody titer correlates positively with protection (reviewed in [74]). However, the role of neutralizing antibodies in protection against fetal infection during pregnancy is complex. Placental infection may occur prior to 14 dpi, suggesting the maternal immune response may not have time to develop in time to protect the fetus [26,27]. Further, the administration of neutralizing antibodies at peak viremia to infected pregnant NHPs did not protect against fetal ZIKV infection or virus detection in amniotic fluid, despite clearing the virus from maternal sera [75]. Neutralizing antibodies of maternal origin have been detected in the amniotic fluid and fetal serum at birth, recapitulating what has been observed in humans [22,23,27,76,77,78]. Notably, antibody-mediated protection of the fetus does seem to be limited. At birth, NHP infants born to dams infected with ZIKV during pregnancy have higher levels of inflammatory cytokines in their peripheral blood, cerebrospinal fluid, and amniotic fluid [27]. Maternal antibodies have also been hypothesized to exacerbate fetal infection through antibody-dependent enhancement (ADE). ADE, in which virus uptake and infection are increased via the interaction of cellular Fcγ receptors with an antibody-bound (but not neutralized) virus, has been most prominently demonstrated during sequential infection with heterologous serotypes of dengue virus [79]. ADE of ZIKV infection has also been demonstrated with anti-DENV antibodies in vitro and in mice [80], although not in NHPs or humans [81,82,83,84,85]. However, the ability of maternal antibodies to enhance ZIKV infection in culture demonstrated a positive correlation between enhancement titers and adverse pregnancy outcomes in humans and NHPs [86], suggesting that placental FcRn receptors may mediate increased uptake of the virus. Due to this possibility, the potential for ADE of fetal infection will need to be considered in all future vaccine research. 

## 5. Conclusions and Future Directions

In summary, research efforts made by a number of groups over the past five years have advanced our knowledge of ZIKV infection during pregnancy, with NHP models providing important clinically relevant translational data, and supporting data generated from small animal models and in vitro culture systems. Collectively, it has been well demonstrated that the timing of infection is a key determinant of outcomes and that some cell types are more vulnerable than others to viral transmission. Importantly, ZIKV infects the placenta, leading to altered structure and impeded maternal–fetal exchange capacity. The subsequent impact on fetal development manifests as a range of phenotypes with differing severity, which are broadly classed as congenital Zika syndrome. The next research priority area must focus on therapeutic interventions to mitigate the adverse effects of ZIKV, and the development of vaccine therapies to prevent maternal–fetal viral transfer.

## Figures and Tables

**Table 1:** Infectious agents with known teratogenic potential.

Organism	Mechanism of Infection	Histologic Findings in the Human Placenta	References
Toxoplasma gondii	Transplacental infection occurs with primary infection of the mother; while transmission to the fetus is more common later in gestation, it is more severe in early gestation.	Early spontaneous abortion villous tissues: delayed villous maturation with Hofbauer cell hyperplasia. Later placental tissues: in addition to delayed villous maturation with Hofbauer cell hyperplasia, lymphohistiocytic chronic villitis, which may also be granulomatous and necrotizing. Pseudocysts and true cysts can be seen in the stroma of the cord and chorionic plate. Free tachyzoites may be identified in areas of active inflammation.	[37,51,52]
Treponema pallidum	Transplacental infection is most common in untreated secondary syphilis. Penicillin treatment is thought to be able to prevent 98% of vertical transmission in deliveries >20 weeks.	Classical triad: (1) delayed villous maturation with Hofbauer and stromal cell hyperplasia; (2) thickened fetal vasculature with prominent endo- and perivascular connective tissue; and (3) acute and/or chronic villitis, variably associated with necrosis. Other findings may include necrotizing funisitis, acute chorioamnionitis, and plasma cell deciduitis.	[41,51,53]
Human immunodeficiency virus	Vertical transmission occurs mostly during delivery; however, up to 30% of congenital HIV may be transplacental.	Early therapeutic abortion villous tissues with detection of HIV in fetal tissues: acute chorioamnionitis, plasma cell deciduitis, and necrotizing deciduitis. No specific findings in later placental tissues.	[51,54]
Zika virus	Transplacental infection occurs with primary infection of the mother.	Reports consistently mention Hofbauer cell hyperplasia. Placental infarcts, villous stromal calcifications, and plasma cell deciduitis with leukocytoclastic/lymphocytic vasculitis have been described in rhesus macaques.	[24,27,43,45,51,55,56,57,58]
Varicella zoster virus	Transplacental infection is rare (believed to be <1%) but may occur with primary infection of the mother.	Not well established, but the literature includes a case report describing diffuse, necrotizing chronic villitis with granulomatous inflammation.	[51,59]
Coxsackievirus	Transplacental infection.	Massive perivillous fibrin deposition with trophoblast necrosis and mixed acute and chronic inflammation within the fibrinoid has been described in placentas from stillbirths due to Coxsackievirus A.	[38,51]
Parvovirus B19	Transplacental infection.	The placenta is notable erythroblastosis fetalis in the villous circulation. Characteristic nuclear enlargement and ground-glass inclusions may be striking.	[51]
SARS-CoV-2	Transplacental infection is rare.	Placental pathology may vary depending on whether there is maternal infection only (nonspecific findings reported include maternal and fetal vascular malperfusion) vs. infection of both the mother–baby dyad (chronic histiocytic intervillositis and massive perivillous fibrin deposition, which may co-occur).	[60,61]
Rubella virus	Transplacental infection <16 weeks leads to congenital rubella syndrome (deafness, eye abnormalities, and congenital heart disease).	Fetal vasculitis with necrotizing acute and chronic villitis. Intranuclear and cytoplasmic nuclear inclusions can be seen in various compartments (amnion, endothelial cells, extravillous trophoblast, and decidua).	[39,51]
Cytomegalovirus	Transplacental infection earlier in gestation may lead to fetal hydrops and demise. Congenital cytomegalovirus infection is one of the most common causes of microcephaly and sensorineural hearing loss.	Chronic villitis, characteristically lymphoplasmacytic. Villous stromal hemosiderin deposition is also associated with CMV placentitis.	[40,51]
Herpes simplex virus	Both transplacental and ascending infections involving the amniotic fluid are documented.	Transplacental infection: plasma cell villitis and necrotizing deciduitis. Ascending infection: acute and chronic chorioamnionitis and necrotizing funisitis, with plasma cells in the membranes and cord. HSV viral cytopathic effect may be present.	[51,62]

## Data Availability

Not applicable.
